# Comparative Analysis of Physicochemical Characteristics, Nutritional and Functional Components and Antioxidant Capacity of Fifteen Kiwifruit (*Actinidia*) Cultivars—Comparative Analysis of Fifteen Kiwifruit (*Actinidia*) Cultivars

**DOI:** 10.3390/foods9091267

**Published:** 2020-09-10

**Authors:** Hexin Zhang, Qinyu Zhao, Tian Lan, Tonghui Geng, Chenxu Gao, Quyu Yuan, Qianwen Zhang, Pingkang Xu, Xiangyu Sun, Xuebo Liu, Tingting Ma

**Affiliations:** 1 College of Food Science and Engineering, College of Enology, Northwest A&F University, Yangling 712100, China; zhanghexin1217@nwafu.edu.cn (H.Z.); zqy@nwafu.edu.cn (Q.Z.); lt771451884@nwafu.edu.cn (T.L.); gth280187328@aliyun.com (T.G.); 2018013406@nwafu.edu.cn (C.G.); y23933@nwafu.edu.cn (Q.Y.); sunxiangyu@nwafu.edu.cn (X.S.); xueboliu@nwsuaf.edu.cn (X.L.); 2 Department of Plant and Soil Sciences, Mississippi State University, Starkville, MS 39762, USA; qz72@msstate.edu; 3 Department of Chemistry, College of Science, Food Science and Technology Programme, National University of Singapore, Singapore 119077, Singapore; e0220150@u.nus.edu

**Keywords:** kiwifruit, flesh color, species, physicochemical characteristics, nutritional and functional components, antioxidant capacity

## Abstract

Physicochemical characteristics, nutritional and functional components, and the antioxidant capacity of 15 kinds of domestic and imported kiwifruit in China were studied. Kiwifruit was classified according to flesh color or species, and the differences were analyzed and compared. Results demonstrated Ruiyu had the highest sugar-acid ratio, and Hongshi No.2 was an excellent cultivar with strong antioxidant capacity. TPC (total polyphenol content) and AAC (ascorbic acid content) showed a significant positive correlation. TPC was the greatest antioxidant contributor in the DPPH and FRAP assays. The sugar–acid ratio and TFC (total flavonoids content) in red-fleshed kiwifruit were significantly higher than those in yellow-fleshed and green-fleshed ones. The composition of free amino acids had a tendency to distinguish *A. deliciosa* and *A. chinensis*, but this needs further verification. In addition, the contents of mineral elements, folic acid and L-5-methyltetrahydrofolate were also analyzed. Generally, kiwifruit contains comprehensive nutrients and has strong antioxidant capacity. Cultivar is one of the main factors affecting nutritional and functional properties and antioxidant capacity.

## 1. Introduction

*Actinidia*, the basal genus belonged to Actinidiaceae, well-known as kiwifruit due to its association with New Zealand, is actually native to China. Over 70 species have been developed gradually all over the world through decades of domestication and constant selection from wild kiwifruit. Among these, 62 species are naturally distributed in China [1,2]. Kiwifruit is cultivated in temperate areas with a latitude between 25° and 45°. China, Italy, New Zealand, Chile, and Greece are the top five producers in the world, accounting for 87% of the total yield of kiwifruit [3]. Specifically, in China, kiwifruit cultivation is mainly distributed in Shaanxi, Sichuan, Chongqing, Guizhou, Hunan, Hubei, Henan, and other provinces and regions, and the yield from Shaanxi and Sichuan accounts for nearly 80% of the total. Among the large number of kiwifruit species, only a few have been developed extensively because of their admirable flavor and important commercial value, including *A. deliciosa* and *A. chinensis* [3]. The hair of *A. deliciosa*’s rough skin is long, hard, and with late abscission. In contrast, the hair of *A. chinensis*’s smooth skin is short, soft, and with nearly complete abscission when it is ripe. Generally, the color of most kiwifruit flesh is green, yellow or red (the middle part) at harvest, depending on the type and quantity of pigments. *A. deliciosa* has green flesh when ripe, whereas *A. chinensis* often has yellow flesh when ripe [4,5].

Excellent flavor and outstanding nutritional quality are the main reasons why kiwifruit is widely accepted by consumers, and it is value-added in the fruit market. The flavor of kiwifruit is related to its aroma, taste (sugar, acid) and texture [6,7]. There are comprehensive and independent data regarding the nutritional composition of kiwifruit in the USDA National Nutrient Database for Standard Reference and the New Zealand Food Composition Database [8]. Vitamin C, also known as ascorbic acid, is one of the most unique nutritional properties in kiwifruit. Taking Sungold as an example, the content of vitamin C is almost three times that of orange and strawberry (calculated by fresh weight). It enjoys a reputation as “the king of Vitamin C” in China [3,9]. Kiwifruit is also rich in polyphenols, flavonoids, anthocyanins and other antioxidant ingredients [10,11,12]. Generally, phytochemicals are regarded to be the most important bioactive components of kiwifruit [11]. It is a trend that synthetic antioxidants are being replaced by naturally occurring antioxidants in food industry [13]. It has previously been observed that kiwifruit extract is a natural antioxidant, which exhibited strong inhibitory effects on HepG2 and HT-29 cell growth [14,15]. Data from several studies have suggested that eating kiwifruit could minimize the symptoms of chronic diseases, such as hyperlipidemia, diabetes, and inflammatory diseases [16]. Recent evidence has suggested that kiwifruit polyphenols which are rich in anthocyanin could modulate key inflammatory signals (CCL11 and NF-κB) and have potential benefits in reducing lung inflammation [10]. In addition, kiwifruit is also a good source of carotenoids, vitamin E, amino acids, mineral elements, folate, and other nutrients. Particularly, folate is easily damaged in the cooking process, so fresh-eating is the best way to obtain it [8,17,18,19,20].

A deep understanding of flavor and nutrition will promote consumers to select cultivars of kiwifruit properly and produce a higher market value for the cultivation of new cultivars, which is crucial for the production and consumption of kiwifruit. Nevertheless, there is an imbalance between low economic effectiveness and high kiwifruit production. The imported kiwifruit has more market advantages than the local ones [21]. To date, there are few studies that have investigated the association between nutritional value, functional characteristics and flesh color of kiwifruit cultivars or species, and the sample sizes are small as well.

In this study, 15 kinds of classic and new cultivars emerging in China in recent years, including three flesh colors and two commercially valuable species, were selected to supplement some nutritional, functional and antioxidant indexes, such as free amino acids and folates, which are frequently ignored. On the basis of their properties, kiwifruit cultivars with different flesh colors or different species were analyzed to find the rule, in order to supply some data about the nutritional and functional components of kiwifruit in Chinese market to help consumers select kiwifruit and promote the economic return.

## 2. Materials and Methods

### 2.1. Samples and Chemicals

**Figure 1:**
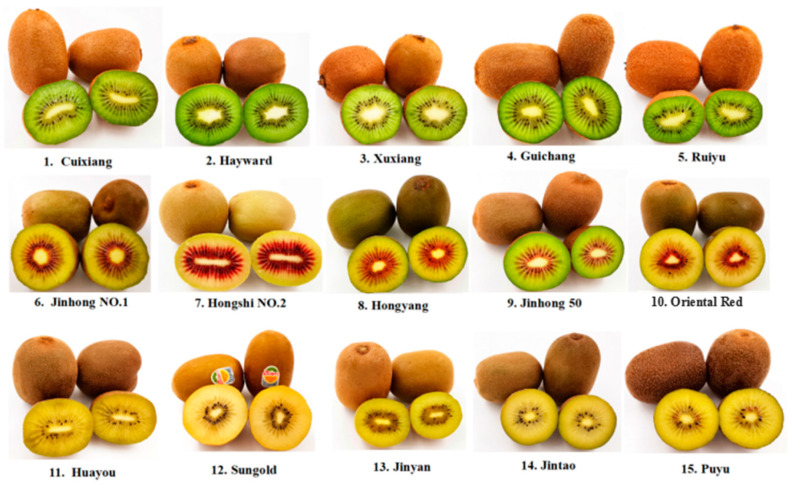
Sample photos of 15 kiwifruit (*Actinidia*) cultivars.

The 15 kiwifruit cultivars were purchased from production bases and retail markets in several provinces rich in kiwifruit. Except for Sungold, which was imported from New Zealand, and Hayward, which was brought in from Zealand and has been widely cultivated in China, other cultivars were developed and widely grown in China. The kiwifruits used in this experiment harvested in 2019 were all at commercial maturity, and their hardness was between 3.6–6.7 N/cm^2^. Relevant sample information was shown in Figure 1 and Table 1. Two kilograms (kg) of each kiwifruit cultivar were peeled, cut into pieces, homogenized, and sealed sequentially. The homogenate was weighed directly to measure TSS (total soluble solids) and pH. Meanwhile, TA (titratable acid), AAC, TPC, TFC, and TAC (total anthocyanins content) were determined through extracts of the homogenate at the same time. When measuring other components, the homogenate was prepared instantly before determination. The chemicals used in the experiments were of analytical grade or high-performance liquid chromatography grade.

### 2.2. Total Soluble Solids (TSS), Titratable Acid (TA), pH

The TSS were determined using homogenate by a PAL-1 Abbe Refractometer (ATAGO, Tokyo, Japan) based on Chinese National Standards (CNS) NY/T 2637-2014 [22]. The preparation of the TA extract and the determination of TA using an acid-base titration method were based on CNS GB/T 12456-2008 [23]. The pH values were measured using homogenate by a PHS-3E pH meter (Shanghai Leici Co. Ltd., Shanghai, China) based on Chinese National Standards GB/T 10468-1989 [24] and the previous study [25].

### 2.3. Ascorbic Acid Content (AAC), Total Polyphenol Content (TPC), Total Flavonoids Content (TFC), Total Anthocyanins Content (TAC)

#### 2.3.1. Extracts

AAC extract: 5.0 g of kiwifruit homogenate and 45 mL of 2% oxalic acid solution were mixed and rested for 1 h, and the supernatant was transferred to a 100 mL volumetric flask after centrifuging, and diluted with oxalic acid solution. The AAC extract concentration was 50 mg/mL.

Polyphenol extract: 5.0 g of homogenate was mix with 20 mL of 70% ethanol solution, ultrasonic extracted for 15 min and centrifuged at 4 °C 8000 r/min for 10 min to obtain the supernatant. The above operation was repeated once for the precipitate, and the supernatant was combined. The supernatant was the crude polyphenol extract for the determination of TPC, TFC, and TAC. The polyphenol extract concentration was 100 mg/mL.

The L*, a*, b*, and ΔE values of the extract were determined with a Ci7600 colorimeter (X-rite, Grand Rapids, USA, Michigan) according to GB/T 6324.6-2014 [26].

#### 2.3.2. Determination

The AAC was determined using a 2,6-dichloro-indophenol titration method based on CNS GB 5009.86-2016 [27]. The TPC was determined by Folin-Ciocalteu colorimetry as follows: 0.2 mL of the polyphenol extract was added in 2 mL of the diluted Folin-Ciocalteu solution and 1.8 mL of 7.5% sodium carbonate solution, then mixed and placed in the dark for 1 h, the absorbance was measured at 765 nm. And the results were expressed as mg gallic acid equivalents (GAE)/100 g flesh weight (FW) [28,29]. The TFC was determined as described previously as follows: 0.5 mL of polyphenol extract and 0.3 mL of 0.5 mol/L sodium nitrite solution were mixed and rested for 3 min, then 0.3 mL of 0.3 mol/L aluminum chloride solution, 2 mL of 1 mol/L sodium hydroxide solution, and 70% ethanol to 5 mL were added sequentially and rested for 10 min, the absorbance was measured at 506 nm. The results were expressed as mg catechin equivalents (CTE)/100 g flesh weight (FW) [30]. The TAC was estimated using the pH differential method as follows: 0.2 mL polyphenol extract was taken twice, 3.8 mL pH = 1 hydrochloric acid-sodium chloride buffer solution was added to the first solution, and 3.8 mL pH = 4.5 acetic acid-sodium acetate buffer solution was added to the second solution, and the absorbance of the two solutions was measured at 510 nm and 700 nm. The results were expressed as mg cyanidin-3-glucoside (CGE)/100 g flesh weight (FW) [30].

### 2.4. Analysis of Antioxidant Capacity

This study used two different methods to estimate antioxidant capacity, 1,1-diphenyl-2-picrylhydrazyl (DPPH) and ferric reducing antioxidant power (FRAP), which were slightly modified from previous studies [21,30]. DPPH determination was as follows: 0.25 mL polyphenol extract (or TAA extract) in Section 2.3.1 and 4 mL freshly prepared DPPH solution reacted for 30 min in the dark, and the absorbance was measured at 517 nm. FRAP determination was as follows: 0.25 mL of polyphenol extract (or TAA extract) in Section 2.3.1 and 8 mL of TPTZ (tripyridyltriazine) working solution reacted for 10 min in a 37 °C water bath, then the absorbance was measured at 593 nm. The results are expressed as μmol trolox/g FW.

### 2.5. Protein and Free Amino Acids

The protein content was determined according to CNS GB 5009.5-2010 [31] using the Kjeldahl method as follows: 2.0 g of homogenate was added 0.7179 g of copper sulfate pentahydrate, 6.2821 g of potassium sulfate, and 10 mL of concentrated sulfuric acid sequentially. Then, it was decomposed at 420 °C for 1 h until being clarified. It was tested by a Kjeltec 2300 automatic analyzer (FOSS, Hillerød, Denmark).

The concentration of free amino acids was determined according to the national standard GB 5009.124-2016 [32], and some modifications were made as follows: 2.0 g homogenate was added 10 mL 6 mol/L hydrochloric acid with ultrasonic processing for 2 min, and it was put in a stove at 110 °C for 22 h. Then, it was diluted with ultrapure water 500 times, and filtered with a 0.45 inorganic filter membrane to be determined by using the Hitachi L-8900 amino acid analyzer (Hitachi, Tokyo, Japan).

### 2.6. Analysis of Mineral Elements

Three grams of the homogenate was weighed into an Erlenmeyer flask, 10 mL of nitric acid and 0.5 mL of perchloric acid were added and digested on an adjustable heating plate. After a pretreatment with nitric acid or perchloric acid wet digestion, the PinAAcle900 Atomic Absorbance Spectrophotometer (PerkinElmer Limited, Waltham, MA, USA) was used to detect these mineral elements.

Each mineral element was determined by the national standard method as follows: Ca (GB 5009.92-2016) [33], K, Na (GB 5009.91-2017) [34], Cu (GB 5009.13-2017) [35], Zn (GB 5009.14-2017) [36], Mg (GB 5009.241-2017) [37], P (GB 5009.87-2016) [38], and Fe (GB 5009.90-2016) [39].

### 2.7. Analysis of Folic Acid (FA) and L-5-Methyltetrahydrofolate (5MTHF)

The measurement was performed by HPLC and the method according to Czarnowska-Kujawska et al. [40] was slightly modified as follows. Thereby, 8 g of kiwifruit homogenate was added 20 mL of 0.1 M phosphate buffer to the centrifuge tube, and it was blown on a nitrogen blower for 15 s, then put in a 90 °C water bath for 5 min. It was put in an ice bath under dark conditions and centrifuged. The supernatant was pass through an inorganic filter membrane for testing. Gradient elution conditions: A Waters Alliance 2695 HPLC system with a 2996 PDA Detector (Waters Corp., Milford, MA, USA) was used to simultaneously separate and analyze the FA and 5MTHF. The system was run at 0.4 mL/min using a HC-C18 column (2) (Agilent, Santa Clara, CA, USA 250 × 4.6 mm, 5 μm). The column temperature was set at 25 °C, and 20 μL of sample was injected. The detection wavelength was 285 nm. The mobile phase was phosphate buffer and acetonitrile. The condition was that 6% acetonitrile combined 94% phosphate buffer in first 5 min, in 5–25 min linear change was from the original ratio to 25% acetonitrile combined 75% phosphate buffer, then it maintained for 2 min. The flow term was linear in the range of 27–28 min, then changed back to the original concentration ratio.

### 2.8. Statistical Analysis

The experimental results were expressed as the mean ± SD of three parallel measurements, and data analysis was performed using SPSS statistics 23 software (International Business Machines Corporation, Armonk, NY, USA). Multigroup comparisons of the means were carried out by one-way analysis of variance (ANOVA) test with post-hoc contrasts by LSD test and Duncan test. The statistical significance for all tests was set at *p* < 0.05. Using Euclidean distance as a measure of dissimilarity, hierarchical cluster analysis and heat maps were constructed using the data of free amino acids and mineral elements, respectively. The average values of the amino acids and the mineral elements were used as input data in principal component analysis (PCA). The R programming language (Ross Ihaka & Robert Gentleman, Auckland, New Zealand) was used to perform correlation analysis.

## 3. Results and Discussion

### 3.1. Physicochemical Characteristics

#### 3.1.1. Total Soluble Solid (TSS), Titratable Acid (TA) and pH

**Figure 2:**
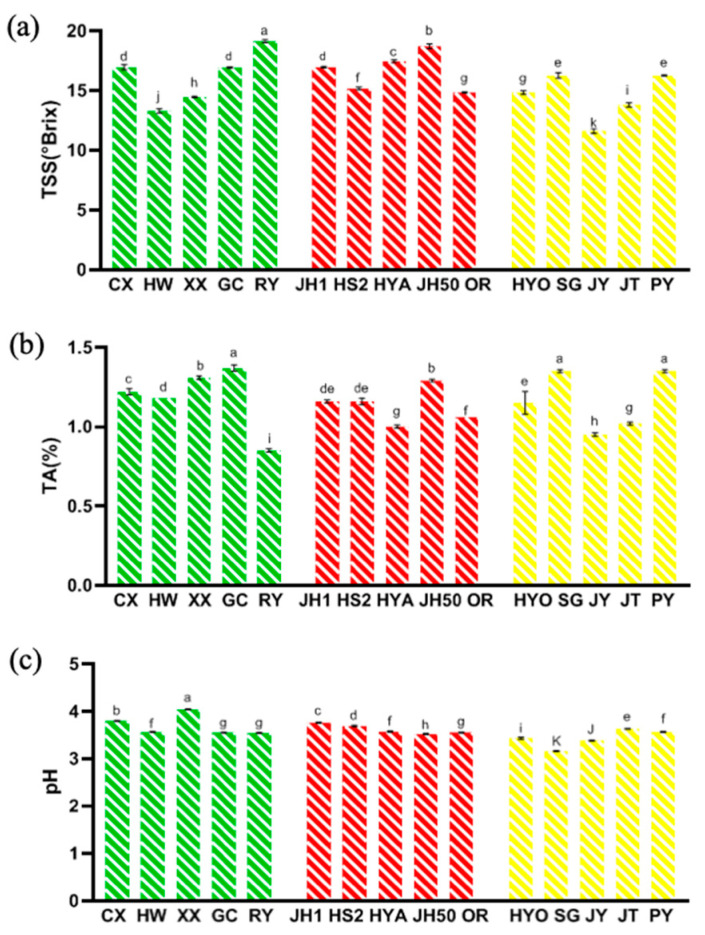
Three physicochemical characteristics in 15 kiwifruit cultivars. (**a**) Total soluble solid (TSS); (**b**) Titratable acid (TA); (**c**) pH (mean ± SD; *n* = 3). Mean values in each column with unlike letters are significantly different among cultivars (*p* < 0.05).

Figure 2 illustrated the physical and chemical characteristics of different kiwifruit cultivars, including TSS, TA and pH. Generally, TSS consists of sugar, acids, vitamins, certain minerals, and other soluble solids, which are essential indicators of sensory quality and consumer recognition [41]. The TSS for all cultivars ranged from 11.60 to 19.13° Brix (Figure 2a), which was larger than the TSS range in previous studies [12]. Among them, the TSS of Ruiyu (19.13° Brix), Jinhong 50 (18.70° Brix) and Hongyang (17.47° Brix) were the highest, and that of Jintao (13.80° Brix), Hayward (13.30° Brix), and Jinyan (11.60° Brix) were the lowest.

Titratable acid (TA) also plays an important role in the taste of fruits. From Figure 2b, the TA of 15 cultivars was between 0.85% and 1.35% (based on citric acid). Compared with other cultivars, Puyu (1.35%), Sungold (1.35%), and Guichang (1.37%) had higher TA values. In addition, the pH of all 15 kiwifruit cultivars was between 3.16 and 4.04 (Figure 2c). The results of TA and pH were consistent with the previous study [41], showing the range of TA from 1.14% to 1.82% and the range of pH from 3.06 to 3.56.

#### 3.1.2. Sugar-Acid Ratio

**Figure 3:**
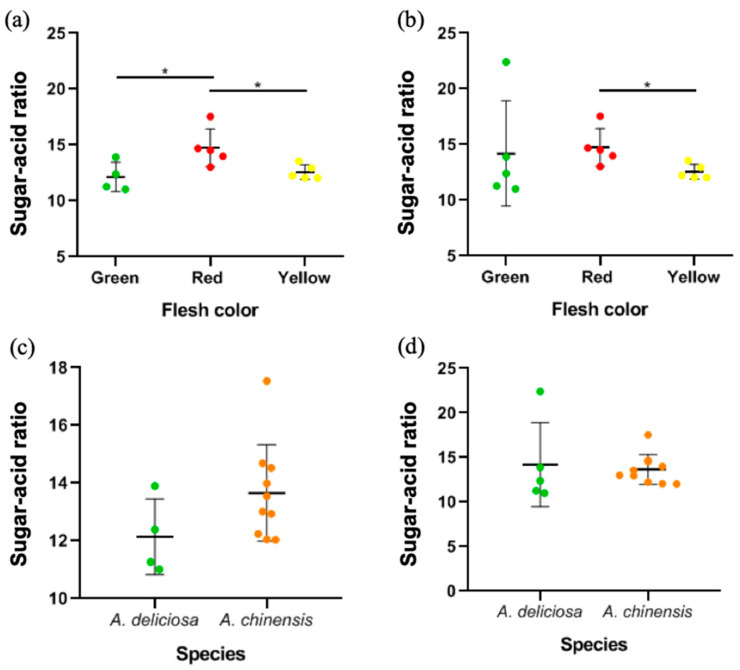
(**a**) The distribution of sugar-acid ratio in different flesh colors of 14 cultivars, which is not included the cultivar “Ruiyu”. (**b**) The distribution of sugar-acid ratio in different flesh colors of 15 cultivars. (**c**) The distribution of sugar-acid ratio in two species of 14 cultivars, which is not included the cultivar “Ruiyu”. (**d**) The distribution of sugar-acid ratio in two species of 15 cultivars. Mean values in each column with “*” are significantly different (*p* < 0.05).

The sugar-acid ratio (TSS:TA), also known as the maturity index, which may influence fruit taste and flavor, is an important parameter for the classification of cultivars [42]. Here, we classified different kiwifruit cultivars according to their flesh color and species for differential analysis. As shown in Figure 3b, the sugar–acid ratio of red-fleshed kiwifruit was significantly higher than that of yellow-fleshed kiwifruit (*p* < 0.05) but not significantly different from that of green-fleshed kiwifruit. However, after removing the cultivar Ruiyu, which had the highest sugar-acid ratio (Figure 3a), the sugar-acid ratio of red-fleshed kiwifruit was significantly higher than that of green-fleshed kiwifruit (*p* < 0.05). In 2017, a national protection right as a new plant cultivar was obtained for Ruiyu. The new cultivar came into being in Shaanxi Province, China for transformation and upgrading of kiwifruits. The sugar-acid ratio of Ruiyu also confirmed its status as a breakthrough; that is, green-fleshed kiwifruit was no longer characterized by a low sugar-acid ratio. As shown in Figure 3c,d, based on the species classification, there was no significant difference between *A. deliciosa* and *A. chinensis* in sugar-acid ratio (*p* > 0.05).

### 3.2. AAC, TPC, TFC, TAC

Table 2 gave information about the nutritional and functional components with antioxidant function for different kiwifruit cultivars, including AAC, TPC, TFC, and TAC. Vegetables and fruits are the lowest-cost sources of AAC, and AAC is a priority factor in the evaluation of many fruits [43]. In this study, AAC ranged from 52.39 to 248.16 mg/100 g, and it was very high in Hongshi No.2 (248.16 mg/100 g), Puyu (233.99 mg/100 g), Huayou (187.33 mg/100 g), and Oriental Red (181.07 mg/100 g). The results of AAC in this study were similar to those in the study of Li et al. (2010) [44], which ranged from 58.3 to 217.1 mg/100 g.

**Figure 4:**
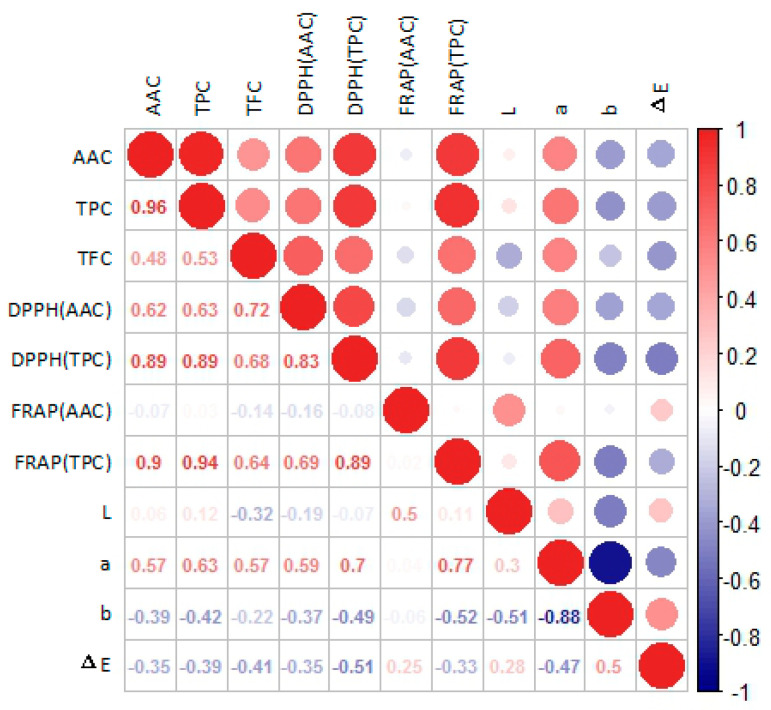
Correlation heat map of the antioxidant analysis.

Phenols are a class of compounds influencing a variety of plant properties, such as color, bitterness, and antioxidant activity [15]. As shown in Table 2, TPC, TFC, and TAC were also significantly different among the different cultivars. In this study, the TPC of different kiwifruit cultivars ranged from 74.06 to 216.37 mg GAE/100 g, which was quite wider than the result in previous studies. As Cozzolino et al. [7] reported, the TPC of five cultivars ranged from 83.1 to 169.0 mg GAE/100 g. For the cultivar “Jintao” which was the same cultivar as samples in both studies, the TPC was about 124 mg GAE/100 g in the study of Cozzolino et al., and was about 141.7 mg GAE/100 g in this study. Interestingly, the cultivars with the four highest levels of TPC showed the same trend for AAC. In fact, in subsequent antioxidant correlation analysis (Figure 4), TPC was significantly correlated with the content of AAC (*R*^2^ = 0.96). These conclusions were consistent with our previous research [21]. Flavonoids are usually considered to be effective antioxidants [14]. In this study, TFC ranged from 5.69 to 50.89 CTE mg/100 g, and for Hongshi No.2 (50.89 CTE mg/100 g), Hongyang (40.69 CTE mg/100 g), and Jinhong 50 (31.64 CTE mg/100 g), the TFC content was significantly higher than that of the other cultivars (*p <* 0.05). Compared with the result in the study of Du et al. [28], where the range of TPC was from 6.64 to 74.24 mg/100 g, the result of TFC in this study was lower due to less species probably. We found that the TAC in green-fleshed and yellow-fleshed kiwifruit was almost undetectable, and the error for measurement was relatively large, so those values were not shown in the table. This finding was consistent with a recent study, in which almost no anthocyanin was detected in green-fleshed and yellow-fleshed kiwifruit [10]. In this study, Hongshi No.2 had the highest AAC, TPC, TFC, and TAC among the 15 kiwifruit cultivars. Obviously, Hongshi No.2 has an outstanding content of the four antioxidant-related compounds, and therefore, Hongshi No.2 is an excellent cultivar with strong antioxidant capacity.

**Figure 5:**
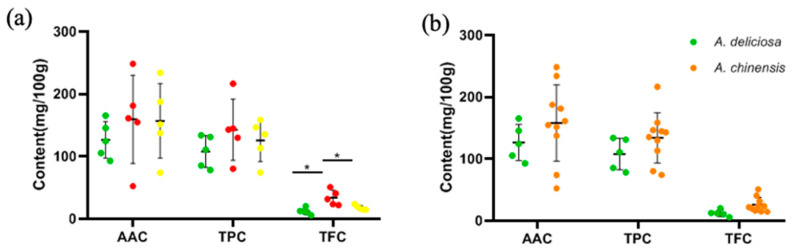
The distribution of AAC, TPC and TFC (**a**) in different flesh colors and (**b**) in two species. The colors of the dots in (**a**) are consistent with flesh color.

According to the difference analysis of AAC, TPC and TFC based on flesh color classification, as shown in Figure 5a, only the difference in TFC was statistically significant (*p* < 0.05), but there were no significant differences in AAC and TPC (*p* > 0.05). Overall, the TFC in red-fleshed kiwifruit was significantly higher than in green-fleshed and yellow-fleshed kiwifruit. In addition, there was no significant difference based on species classification (*p* > 0.05) (Figure 5b).

### 3.3. Antioxidant Activities

The antioxidant capacity of kiwifruit in vitro was determined by examining its ability to eliminate free radicals [8]. According to relevant reports, the total antioxidant capacity of kiwifruit was higher than that of apples, grapefruits, and pears, but lower than that of raspberries, strawberries, and oranges [45]. In this study, we used the oxalic acid extract of kiwifruit, which was used to measure AAC, and the phenolic alcohol extract, which was used to measure TPC, TFC, and TAC, in order to perform the DPPH and FRAP assays, respectively. A comparison of these two extracts was not available in previous studies, so we aimed to analyze the antioxidative capacity of the polyphenols and AAC, respectively.

As shown in Table 3, in the DPPH assay, the DPPH value of the AAC extract (DPPH(AAC)) was between 1.29 µmol Trolox/g and 5.02 µmol Trolox/g, and the DPPH value of the phenol extract (DPPH(TPC)) was between 3.69 µmol Trolox/g and 11.96 µmol Trolox/g. In the FRAP assay, the FRAP value of the AAC extract (FRAP(AAC)) was between 5.73 µmol Trolox/g and 25.92 µmol Trolox/g, and that of the phenol extract (FRAP(TPC)) was between 6.22 µmol Trolox/g and 17.69 µmol Trolox/g. Overall, among the 15 tested cultivars, Cuixiang showed the best antioxidant capacity among the green-fleshed kiwifruit, and Hongshi No.2 had the best antioxidant capacity among the red-fleshed kiwifruit.

Antioxidant correlation analysis (Figure 4) was used to explore the relationship between different antioxidant variables measured in the kiwifruit extracts. AAC and TPC are the main factors for the antioxidant activity of kiwifruit, and the variables included AAC, TPC, TFC, DPPH (AAC), DPPH (TPC), FRAP (AAC), FRAP (TPC), and the L, a, b, and Δ E values of polyphenol extraction. As mentioned above in Section 3.2, the levels of TPC and AAC in kiwifruit were significantly correlated (*R*^2^ = 0.96), which was similar to the findings of a previous study [21]. The correlation coefficient between DPPH (AAC) and AAC was 0.62, between DPPH (TPC) and TPC it was 0.89, and between DPPH (TPC) and TFC it was 0.68. There was almost no correlation between FRAP (AAC) and AAC, but the correlation coefficient between FRAP (TPC) and TPC was as high as 0.94, and between FRAP (TPC) and TFC it was 0.64. These data mean that in the DPPH assay, AAC, TPC and TFC all contributed to the antioxidant performance of kiwifruit, while the TPC’s contribution was greater. In the FRAP essay, AAC contributed little to the antioxidant capacity, TFC contributed relatively less, while TPC played a dominant role. Additionally, the correlation coefficient between the chroma value “a” for the polyphenol extraction of kiwifruits and DPPH (TPC) was 0.70, and the correlation coefficient between the chroma value “a” and FRAP (TPC) was 0.77. The larger the value of “a”, the redder the extract was, which could indirectly reflect the correlation between red pigment in red-fleshed kiwifruit and its antioxidant capacity.

### 3.4. Total Protein (TP) Content and Total Free Amino Acids (TAA) Content

**Figure 6:**
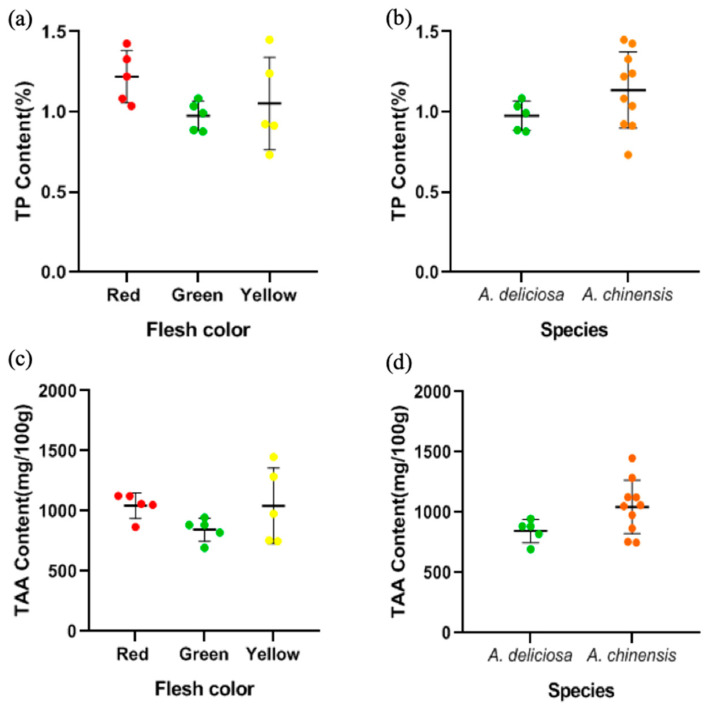
The distribution of TP content (**a**) in different flesh colors and (**b**) in the two species. The distribution of TAA content (**c**) in different flesh colors and (**d**) in the two species.

Protein is a minor but vital component of kiwifruit, and its content of crude protein is approximately 1% of its fresh weight [21,46]. In this study, the protein content of different cultivars of kiwifruit was between 0.73% and 1.45% (Figure 6a), with the highest in Puyu and the lowest in Sungold. The range was consistent with nutritional tables which was typically put the content of protein in kiwifruit at about 1 g/100 g [46]. There were no significant differences between the groups based on species or flesh color analysis (Figure 6a,b) (*p* > 0.05).

In this study, the TAA content of different cultivars of kiwifruit was between 693.37 and 1449.04 mg/100 g (Figure 6c,d), among which the TAA content of Jintao was the highest and that of Hayward was the lowest. The TAA content was not significantly different between different flesh colors and different species (*p* > 0.05) (Figure 6c,d), but there were great differences between different cultivars or even different species in free amino acid composition (Table 4). Overall, the Glu content in all cultivars was at a high level between 109.82 and 237.24 mg/100 g. For most cultivars except Xuxiang and Cuixiang, Arg content showed an excellent performance, especially in Jintao at up to 318.57 mg/100 g. On the other hand, the Met and Pro content were at a low level, less than 10 mg/100 g. In addition, the contents of the other amino acids were relatively moderate, around tens of mg/100 g.

**Figure 7:**
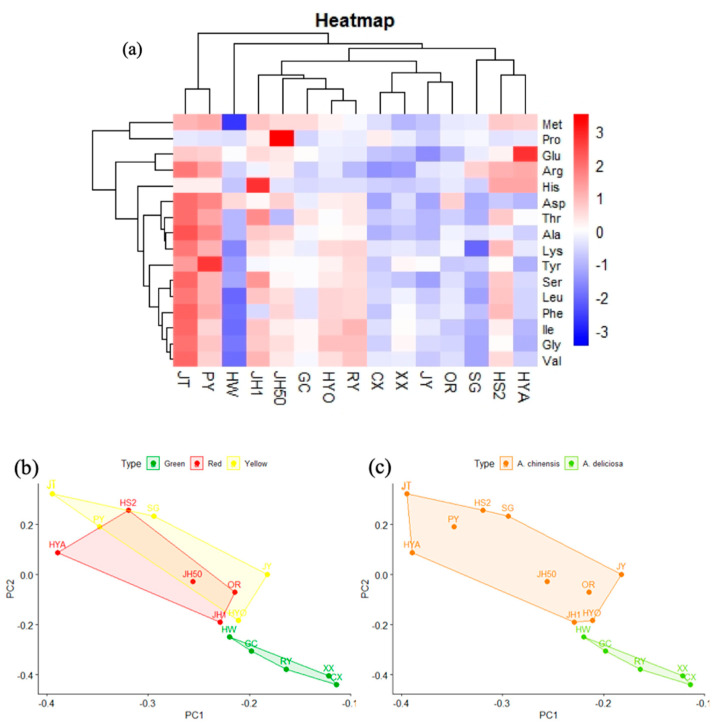
Preliminary analysis of amino acid data. (**a**) Cluster heat map of amino acids in 15 kiwifruit cultivars. (**b**) PCA score plot of amino acids in different flesh colors. (**c**) PCA score plot of amino acids in two species.

As shown in Figure 7a, the cluster heatmap divided the 15 kiwifruit cultivars into five categories. The first category contained Jintao and Puyu (both *A. chinensis*), and with the exception of Pro, their contents of other amino acids were above average. The second category contained Hongyang, Hongshi No.2 and Sungold (all *A. chinensis*), and their contents of Met, Arg, and His were above average. The third category contained Jinhong No. 1, Jinhong 50, Guichang, Huayou, and Ruiyu, which had higher than average amino acid contents of Ile and Gly. The remaining kiwifruit cultivars were in the fourth and fifth categories (Hayward), with low total amino acid content, and most of their free amino acids were also at low levels. Figure 7b,c showed the results of the PCA of the amino acids in different cultivars of kiwifruit and it revealed that the different groups could not be well distinguished based on flesh color. Meanwhile, based on species, the two tended to be distinguished, from which it could be inferred that species is still the main factor affecting free amino acid content. Overall, kiwifruit is a source of almost all nonessential and essential amino acids, which is a manifestation of its nutritional balance.

### 3.5. Mineral Elements

Table 5 lists the contents of eight mineral elements, including Ca, Fe, K, Mg, Na, P, Zn, and Cu, in the different cultivars of kiwifruit. Overall, kiwifruit contained a large amount of K (212.5–516.67 mg/100 g) and an extremely low content of Na (4.68–11.22 mg/100 g). High sodium intake has been associated with high blood pressure, kidney dysfunction, asthma, stomach cancer and osteoporosis. On the contrary, consumption of potassium from fruits and vegetables is benefit to lower blood pressure and can counteract the negative effects of sodium on blood pressure. [47] It is not hard to see that kiwifruit is a typical kind of fruit with high potassium and low sodium, which can reduce the risk of the diseases mentioned above. In addition, kiwifruit is a favorable source of Mg (29.55–60.4 mg/100 g), Ca (8.74–31.27 mg/100 g), P (10.5–38.29 mg/100 g) and Fe (2.71–13.75 mg/100 g). According to the data from a previous study, compared with citrus fruits such as orange, pomelo, mandarin, lemon, and grapefruit, kiwifruit has two to five times as much potassium and nearly three times as much magnesium as these citrus fruits [21,48].

**Figure 8:**
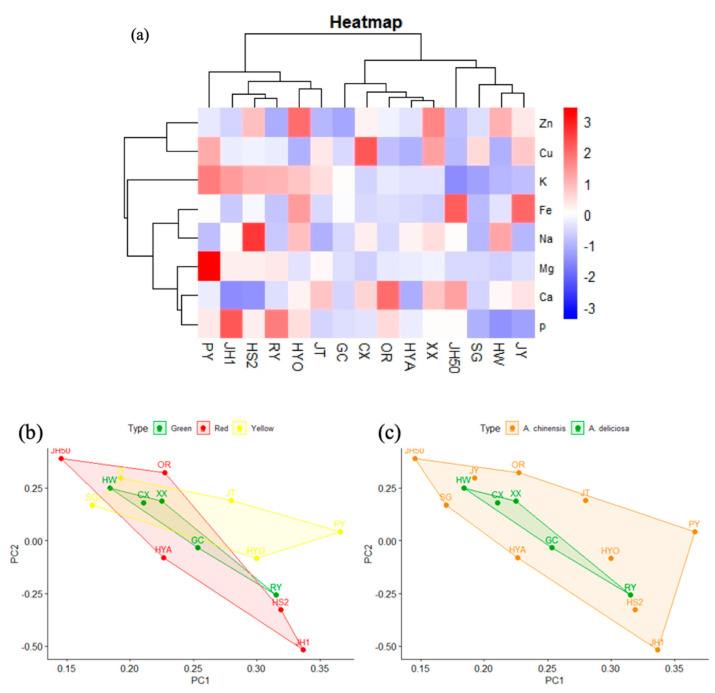
Preliminary analysis of trace element data. (**a**) Cluster heat map of trace elements in 15 kiwifruit cultivars. (**b**) PCA score plot of trace elements in different flesh colors. (**c**) PCA score plot of trace elements in two species.

Similarly, cluster analysis of the above eight mineral elements in the 15 kiwifruit cultivars (Figure 8a) found the kiwifruits could be divided into three categories. The first one contained Puyu, Jinhong No. 1, Hongshi No.2, Ruiyu, and Huayou, which were abundant in K and P. The second category was Jinhong 50, Sungold, Hayward, and Hongshi No.2. The remaining cultivars were in the third category. Principal component analysis based on flesh color and species showed no significant difference (Figure 8b,c), which may be because some of the factors affecting mineral element contents are climatic conditions and soil acidity and alkalinity, regardless of species [21,49].

### 3.6. FA and 5MTHF

Fruits, especially berries and vegetables and green leafy vegetables, are the main sources of folic acid in the diet, of which 5MTHF is the most important folic acid derivative. 5MTHF does not require the complex absorption, metabolism and enzymatic steps required for utilization of FA in the body. However, compared with other nutrients, folic acid data in the literature and databases are not very consistent because, first, the high sensitivity to the environment and the low concentration of folic acid in the biological material, and second, the methods used for extraction and determination of folic acid are different [21,40].

**Figure 9:**
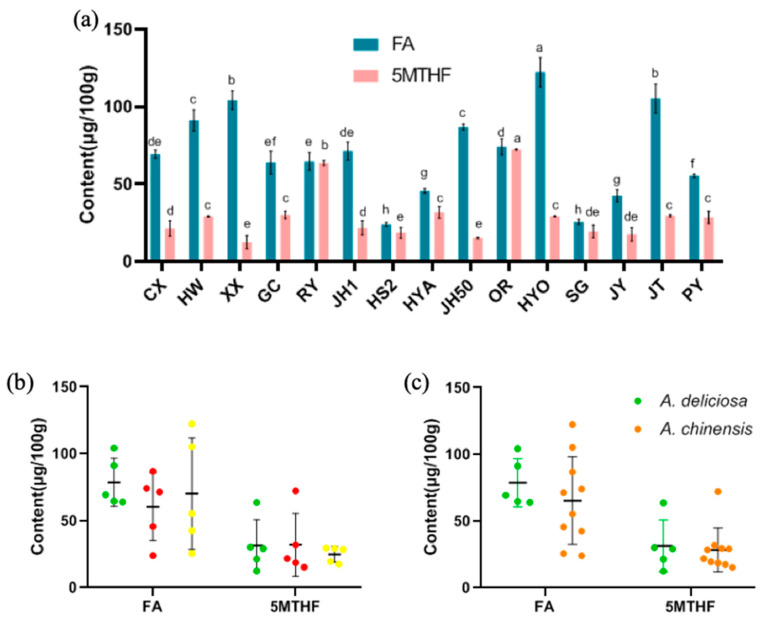
(**a**) The content of FA and 5MTHF in 15 kiwifruit cultivars (mean ± SD, *n* = 3). Mean values in each column with unlike letters are significantly different among cultivars (*p* < 0.05). The distribution of FA and 5MTHF (**b**) in different flesh colors and (**c**) in the two species. The colors of the dots in (**b**) are consistent with the flesh color.

In this study, the content of FA in the different cultivars of kiwifruit was between 23.99 and 122.25 μg/100 g and 5MTHF was between 12.48 and 72.22 μg/100 g (Figure 9a). The contents of FA in Huayou (122.25 μg/100 g), Jintao (105.14 μg/100 g) and Xuxiang (104.14 μg/100 g) were the highest, and meanwhile, the contents of 5MTHF in Oriental Red (72.22 μg/100 g), and Ruiyu (63.58 μg/100 g) were significantly higher than in the other cultivars (*p <* 0.05). Taking Sungold as an example, in a previous study, its content of FA was 30.64 μg/100 g, which was similar to the results in this study [19,21]. For 5MTHF, according to previous studies, the content in strawberries was 36–79 μg/100 g and that in citrus fruit was 13–27 μg/100 g. For kiwifruit, the content of 5MTHF was approximately 23 μg/100 g [21,40,50]. In this study, the range of 5MTHF content in kiwifruit was larger due to use of abundant cultivars, and its average content was higher. We thus concluded that kiwifruit is a sound source of FA and 5MTHF among fruits. Since they are generally eaten fresh, kiwifruit is a favorable way to obtain FA and 5MTHF. In terms of the content of FA and 5MTHF, there were no significant differences based on species or flesh color (*p >* 0.05) (Figure 9b,c).

## 4. Conclusions

Generally, as a comprehensive nutritional supplement, kiwifruit is a good source of AAC, TPC, TFC, mineral elements, TAA, FA, and 5MTHF, and it has excellent antioxidant ability. Overall, one of the main factors affecting the physical and chemical characteristics, nutritional and functional components of kiwifruit is the cultivar. New cultivars developed in recent years may violate the characteristics of the original categories. This may be because cultivated varieties are produced by mutations in primitive complex hybrids, which accumulate over time, producing the varieties that exist today. Moreover, the geographical and environmental factors also have a great influence on the quality of fruits [51].

The results of this study identified several excellent cultivars with different characteristics. The new green-fleshed cultivar Ruiyu broke through the low sugar–acid ratio of green-fleshed kiwifruit. The new red-fleshed cultivar, Hongshi No. 2, had outstanding contents of four antioxidant-related compounds. In addition, several general trends were identified. The sugar–acid ratio and TFC had significant differences between different flesh colors. More specifically, the sugar–acid ratio and the TFC of red-fleshed kiwifruit were significantly higher than green-fleshed and yellow-fleshed ones. The TPC and AAC showed a significant positive correlation as well. Furthermore, the analysis of antioxidant correlation demonstrated that TPC was the greatest contributor in both the FRAP and DPPH assays due to its higher correlation coefficient. In terms of species, *A. deliciosa* and *A. chinensis* tended to be distinguished in their composition of free amino acids, which needs further verification.

## Figures and Tables

**Table 1:** Cultivars, species, flesh color and regions of kiwifruits.

Cultivar	*Abbr.*	Species	Flesh Color	Region
Cuixiang	CX	*A. deliciosa*	Green	Shaanxi, China
Hayward	HW	*A. deliciosa*	Green	Shaanxi, China
Xuxiang	XX	*A. deliciosa*	Green	Shaanxi, China
Guichang	GC	*A. deliciosa*	Green	Guizhou, China
Ruiyu	RY	*A. deliciosa*	Green	Shaanxi, China
Jinhong No.1	JH1	*A. chinensis*	Red *	Jiangsu, China
Hongshi No.2	HS2	*A. chinensis*	Red *	Sichuan, China
Hongyang	HYA	*A. chinensis*	Red *	Shaanxi, China
Jinhong 50	JH50	*A. chinensis*	Red *	Sichuan, China
Oriental Red	OR	*A. chinensis*	Red *	Shaanxi, China
Huayou	HYO	*A. chinensis*	Yellow	Shaanxi, China
Sungold	SG	*A. chinensis*	Yellow	New zealand
Jinyan	JY	*A. chinensis*	Yellow	Henan, China
Jintao	JT	*A. chinensis*	Yellow	Henan, China
Puyu	PY	*A. chinensis*	Yellow	Shaanxi, China

Red * represents the color of the middle part of flesh in kiwifruits. The red flesh color means radial red streaks in the cross section of the core.

**Table 2:** AAC, TPC, TFC and TAC of 15 kiwifruit cultivars.

Cultivar	AAC (mg/100 g)	TPC (mg GAE/100 g)	TFC (mg CTE/100 g)	TAC (mg CGE/100 g)
Cuixiang	145.25 ± 0.75 h	131.03 ± 2.84 e	20.39 ± 3.55 de	-
Hayward	92.75 ± 1.75 l	78.04 ± 3.27 h	10.25 ± 2.13 f	-
Xuxiang	105.42 ± 0.63 k	85.49 ± 2.55 g	12.56 ± 3.51 ef	-
Guichang	165.27 ± 3.25 e	133.94 ± 3.52 e	5.69 ± 2.68 f	-
Ruiyu	124.49 ± 0.89 j	110.57 ± 3.46 f	12.00 ± 1.47 ef	-
Jinhong No.1	52.39 ± 1.88 n	80.09 ± 1.91 h	22.19 ± 3.29 d	0.52 ± 0.07 c
Hongshi No.2	248.16 ± 2.67 a	216.37 ± 0.73 a	50.89 ± 3.02 a	3.65 ± 0.27 a
Hongyang	154.57 ± 1.72 g	129.75 ± 1.59 e	40.69 ± 3.01 b	1.02 ± 0.02 b
Jinhong 50	161.04 ± 1.70 f	142.89 ± 3.78 c	31.64 ± 3.69 c	0.72 ± 0.00 c
Oriental Red	181.07 ± 2.10 d	144.53 ± 0.77 c	23.72 ± 5.71 d	1.58 ± 0.13 b
Huayou	187.33 ± 1.88 c	146.46 ± 2.43 c	23.58 ± 1.44 d	-
Sungold	137.42 ± 1.38 i	113.09 ± 3.22 f	16.33 ± 3.06 e	-
Jinyan	73.83 ± 1.28 m	74.06 ± 0.97 h	15.17 ± 1.12 ef	-
Jintao	151.74 ± 1.61 g	141.7 ± 2.19 d	14.69 ± 4.52 ef	-
Puyu	233.99 ± 1.61 b	158.66 ± 2.35 b	19.61 ± 3.26 d	-

The data are given as the mean ± SD (*n* = 3). Mean values in each column with unlike letters are significantly different among cultivars (*p* < 0.05).

**Table 3:** Antioxidant activities of 15 kiwifruit cultivars measured by DPPH and FRAP assays.

Cultivar	DPPH(AAC) (µmol/g)	DPPH(TPC) (µmol/g)	FRAP(AAC) (µmol/g)	FRAP(TPC) (µmol/g)
Cuixiang	3.56 ± 0.3 b	7.88 ± 0.37 c	16.85 ± 0.6 b	9.14 ± 0.46 f
Hayward	2.01 ± 0.09 d	4.87 ± 0.62 f	5.73 ± 0.29 f	7.08 ± 0.91 h
Xuxiang	1.61 ± 0.29 de	5.37 ± 0.44 ef	8.12 ± 0.59 ef	7.62 ± 0.27 g
Guichang	1.29 ± 0.34 e	5.64 ± 0.16 e	11.36 ± 1.49 d	9.7 ± 0.67 ef
Ruiyu	1.7 ± 0.13 de	4.11 ± 0.24 g	13.99 ± 1.63 c	7.8 ± 0.16 gh
Jinhong No.1	1.69 ± 0.06 de	3.69 ± 0.56 g	9.96 ± 1.33 de	8.36 ± 0.12 g
Hongshi No.2	5.02 ± 1.1 a	11.96 ± 0.26 a	8.36 ± 1.1 ef	17.69 ± 0.36 a
Hongyang	2.34 ± 0.29 cd	7.69 ± 0.17 c	7.02 ± 0.38 f	9.81 ± 0.22 ef
Jinhong 50	3.51 ± 0.14 b	8.56 ± 0.48 b	11.05 ± 1.93 de	15.04 ± 0.24 c
Oriental Red	3.87 ± 0.03 b	9.29 ± 0.26 b	9.7 ± 0.61 de	17.41 ± 0.25 a
Huayou	4.05 ± 0.08 b	9.14 ± 0.12 b	10.54 ± 0.27 de	16.38 ± 0.09 b
Sungold	2.65 ± 0.26 c	6.99 ± 0.17 d	25.92 ± 1.81 a	11.71 ± 0.1 d
Jinyan	1.72 ± 0.16 de	4.31 ± 0.13 fg	9.15 ± 1.83 e	6.22 ± 0.27 i
Jintao	1.32 ± 0.32 e	5.86 ± 0.36 e	12.5 ± 1.92 cd	10.05 ± 0.71 e
Puyu	3.64 ± 0.16 b	8.62 ± 0.22 b	6.44 ± 0.19 f	17.66 ± 0.69 a

The data are given as the mean ± SD (*n* = 3). Mean values in each column with unlike letters are significantly different among cultivars (*p* < 0.05).

**Table 4:** Sixteen kinds of amino acids (mg/100 g) in 15 kiwifruit cultivars.

Cultivar	Asp	Thr	Ser	Glu	Gly	Ala	Val	Met	Ile	Leu	Tyr	Phe	Lys	His	Arg	Pro
Cuixiang	57.57 ± 5.41 g	62.64 ± 5.63 d	46.15 ± 0.7 fg	132.85 ± 2.73 g	37.32 ± 0.07 e	48.06 ± 0.87 g	41.31 ± 0.36 ef	4.38 ± 0.66 b	34.81 ± 0.87 ef	46.66 ± 1.04 e	20.39 ± 1.12 ef	28.24 ± 1.24 e	58.62 ± 0.71 f	17.41 ± 1.99 e	43.39 ± 2.23 n	13.6 ± 4.99 b
Hayward	94.67 ± 2.94 cd	57.65 ± 1.53 d	39.25 ± 1.16 h	155.47 ± 8.97 e	22.41 ± 1.1 g	49.28 ± 1.5 g	24.55 ± 0.98 h	1.21 ± 0.13 c	19.31 ± 0.29 h	27.06 ± 0.12 g	13.14 ± 1.28 f	17.86 ± 1.02 f	34.36 ± 0.39 g	14.99 ± 0.21 e	117.37 ± 1.85 j	7.91 ± 0.61 c
Xuxiang	74.37 ± 3.53 ef	65.32 ± 3.32 d	43.72 ± 0.7 g	128.53 ± 1.04 g	46.44 ± 0.17 d	53.49 ± 0.04 fg	42.41 ± 0.16 ef	3.54 ± 0.66 b	39.98 ± 0.17 d	51.37 ± 0.99 d	30.41 ± 0.72 d	34.9 ± 1.75 d	60.12 ± 3.88 ef	16.83 ± 3.5 de	55.74 ± 2 m	9.24 ± 0.36 c
Guichang	79.07 ± 10.76 e	82.48 ± 6.42 c	53.27 ± 0.56 e	157.81 ± 3.32 de	46.33 ± 2.12 d	66.63 ± 2.8 de	43.55 ± 0.72 e	5.73 ± 0.7 3 ab	40.75 ± 0.58 d	48.59 ± 0.41 d	29.11 ± 2.88 d	31.54 ± 3.14 de	61.22 ± 4.26 ef	19.64 ± 3.36 de	111.49 ± 3.86 k	6.4 ± 1.42 c
Ruiyu	90.01 ± 1.65 d	82.1 ± 0.63 c	59.05 ± 0.37 d	146.36 ± 2.47 f	55.53 ± 0.66 b	70.14 ± 0.67 d	52.91 ± 0.21 bc	4.85 ± 0.26 b	49.65 ± 0.29 b	60.6 ± 0.23 c	35.33 ± 1.28 c	39.75 ± 0.88 c	77.81 ± 1.62 c	21.63 ± 1.1 de	90.97 ± 2.08 l	9.42 ± 0.31 c
Jinhong NO.1	86.02 ± 0.94 de	104.06 ± 18 ab	70.91 ± 2.68 b	171.28 ± 2.52 c	54.12 ± 3.43 b	81.42 ± 4.33 c	54.78 ± 2.43 b	6.15 ± 0.92 ab	47.6 ± 2.67 bc	63.8 ± 2.81 b	28.09 ± 3.68 d	30.25 ± 1.42 e	75.93 ± 2.58 c	82.69 ± 5.56 a	152.97 ± 2.01 f	13.74 ± 4.92 b
Hongshi NO.2	68.05 ± 0.47 f	89.85 ± 0 bc	62.53 ± 1.58 c	161.9 ± 4.55 d	45.44 ± 2.66 d	65.21 ± 4.57 e	50.17 ± 3.47 c	5.97 ± 0.92 ab	41.86 ± 2.26 d	63.67 ± 2.96 b	29.9 ± 2.56 d	43.62 ± 3.14 bc	84.61 ± 4.33 b	52.87 ± 2.46 b	252.92 ± 2.7 d	8.06 ± 3.92 c
Hongyang	62.64 ± 0.47 fg	75.37 ± 0.47 c	47 ± 0.79 f	237.24 ± 3.58 a	32.29 ± 0.46 f	55.74 ± 1.1 f	37.76 ± 1.76 f	5.87 ± 2.24 ab	29.64 ± 3.65 f	46.21 ± 2.96 e	16.87 ± 1.28 f	29.79 ± 2.7 e	60.17 ± 1.1 ef	52.72 ± 9.67 b	260.37 ± 1.77 c	9.06 ± 2.34 c
Jinhong 50	97.99 ± 3.41 c	59.66 ± 3.11 d	55.24 ± 1.3 e	165.81 ± 2.15 cd	50.39 ± 0.3 c	79.27 ± 2.48 c	47.68 ± 0.26 d	5.73 ± 0.86 ab	41.98 ± 0.35 d	59.2 ± 0.58 c	28.77 ± 1.28 d	40.78 ± 0.58 c	70.08 ± 2.33 d	22.41 ± 0.69 de	184.54 ± 0.15 e	41.8 ± 1.83 a
Oriental Red	97.25 ± 4.71 cd	64.17 ± 0 d	47.98 ± 4.51 f	128.53 ± 1.14 g	37.5 ± 1.93 e	65.07 ± 5.71 e	39.89 ± 3.31 f	4.57 ± 0.13 b	32.23 ± 2.32 f	49.45 ± 3.59 d	20.89 ± 1.68 e	33.25 ± 1.9 de	63.96 ± 3.75 e	24.06 ± 1.78 d	147.74 ± 1.32 g	10.32 ± 0.76 bc
Huayou	88.68 ± 0.24 d	74.92 ± 4.58 c	57.08 ± 2.88 de	148.85 ± 2.67 f	55.15 ± 2.45 b	68.94 ± 1.65 de	49.66 ± 2.23 cd	5.27 ± 0.59 ab	46 ± 2.55 c	61.3 ± 4.12 c	31.43 ± 3.12 d	40.58 ± 3.65 c	77.58 ± 6.2 c	21.92 ± 1.23 de	139.63 ± 3.47 h	10.25 ± 1.27 bc
Sungold	58.9 ± 5.88 g	56.31 ± 0.37 d	40.5 ± 1.35 h	149.4 ± 1.82 f	30.04 ± 0.73 f	61.98 ± 0.24 e	32.53 ± 0.05 g	4.62 ± 0.07 b	27.63 ± 0.81 g	37.31 ± 1.39 f	21.29 ± 2.72 e	27 ± 2.56 e	26.18 ± 2.01 h	16.98 ± 0.14 e	219.55 ± 0.85 d	10.86 ± 1.22 bc
Jinyan	58.11 ± 4.06 g	57.54 ± 1.68 d	38.56 ± 1.39 h	109.82 ± 3.06 f	38.35 ± 1.19 e	49.81 ± 0.43 g	37.36 ± 0.98 f	3.82 ± 0.53 b	36.49 ± 0.7 e	44.65 ± 0.99 e	29.05 ± 1.04 d	30.3 ± 0.51 e	56.24 ± 1.74 f	19.06 ± 4.6 de	133.86 ± 1 i	5.9 ± 1.73 c
Jintao	125.45 ± 1.88 a	111.58 ± 1.68 a	77.97 ± 0.74 a	178.27 ± 2.14 b	68.46 ± 1.23 a	111.6 ± 0 a	65.72 ± 0.93 a	6.43 ± 0.26 a	60.48 ± 1.1 a	77.33 ± 2.2 a	45.53 ± 0.16 b	54.31 ± 2.63 a	103.89 ± 3.49 a	34.44 ± 3.7 c	318.57 ± 2.77 a	9.03 ± 0.05 c
Puyu	118.21 ± 6 a	98.26 ± 0.32 b	65.39 ± 0.7 c	175.14 ± 0.52 bc	54.66 ± 0.63 b	98.68 ± 0.47 b	51.19 ± 0.16 c	6.62 ± 0.53 a	45.72 ± 1.8 c	66.79 ± 2.03 b	60.87 ± 4.53 a	45.28 ± 2.7 b	87.81 ± 2.84 b	34.44 ± 0.82 c	268.21 ± 1 b	8.16 ± 0.76 c

The data are given as the mean ± SD (*n* = 3). Mean values in each column with unlike letters are significantly different among cultivars (*p* < 0.05).

**Table 5:** 8 kinds of trace elements in 15 kiwifruit cultivars.

Cultivar	Ca (mg/100 g)	Fe (mg/100 g)	K (mg/100 g)	Mg (mg/100 g)	Na (mg/100 g)	P (mg/100 g)	Zn (mg/100 g)	Cu (mg/100 g)
Cuixiang	22.37 ± 2.44 cd	4.27 ± 0.05 cd	300 ± 8.33 h	29.61 ± 0.08 g	6.95 ± 0.1 bc	18.71 ± 0.04 cd	0.3 ± 0.14 ab	1.3 ± 0.13 a
Hayward	19.42 ± 0.36 d	4.8 ± 1.94 cd	262.5 ± 4.17 j	29.55 ± 0.3 g	8.71 ± 1.37 b	10.5 ± 0.42 e	0.36 ± 0.02 ab	0.18 ± 0.02 g
Xuxiang	23.74 ± 3 c	3.83 ± 0.2 d	320.83 ± 12.5 g	33.39 ± 1.16 d	7.33 ± 2.48 bc	21.75 ± 0 c	0.4 ± 0.19 ab	0.98 ± 0.01 b
Guichang	14.95 ± 0.65 e	6.05 ± 1.6 c	358.33 ± 0 f	31.36 ± 0.02 ef	5.62 ± 0.18 c	18.54 ± 1.13 cd	0.2 ± 0.07 ab	0.36 ± 0.01 f
Ruiyu	15.93 ± 0.46 e	3.27 ± 0.02 d	445.83 ± 12.5 c	36.74 ± 0.8 b	5.29 ± 0.16 c	34.83 ± 1 a	0.2 ± 0.1 b	0.47 ± 0.03 e
Jinhong No.1	8.74 ± 0.73 f	3.48 ± 0.06 d	475 ± 8.33 b	36.21 ± 0.14 bc	6.64 ± 0.12 bc	38.29 ± 2.13 a	0.24 ± 0.1 b	0.45 ± 0 ef
Hongshi No.2	9.18 ± 0.48 f	5.78 ± 0.6 c	450 ± 0 c	36.6 ± 0.31 b	11.22 ± 2.86 a	23.67 ± 0.25 bc	0.35 ± 0.19 a	0.48 ± 0.05 e
Hongyang	11.31 ± 0.22 f	4.19 ± 0.42 d	320.83 ± 4.17 g	32.74 ± 0.07 de	6.87 ± 1.46 bc	19.63 ± 4.79 cd	0.26 ± 0.02 a	0.18 ± 0.01 g
Jinhong 50	27.02 ± 1.17 b	13.75 ± 0.88 a	212.5 ± 4.17 l	30.87 ± 0.03 f	6.58 ± 0.66 bc	21.79 ± 0.79 c	0.22 ± 0.04 b	0.23 ± 0.01 g
Oriental Red	31.27 ± 2.65 a	4.53 ± 0.16 cd	325 ± 0 g	32.1 ± 0.15 e	5.62 ± 0.58 c	25.42 ± 5 b	0.27 ± 0.06 ab	0.25 ± 0.02 g
Huayou	19.86 ± 1.68 d	10.87 ± 2.41 b	425 ± 8.33 d	31.46 ± 0.16 ef	8.03 ± 2.05 b	24.83 ± 1 bc	0.43 ± 0.2 a	0.19 ± 0.04 g
Sungold	15.22 ± 1.64 e	2.71 ± 0.03 d	241.67 ± 0 k	30.63 ± 0.07 f	4.73 ± 0.97 c	13.54 ± 0.71 e	0.25 ± 0.09 ab	0.72 ± 0.07 c
Jinyan	21.26 ± 2.5 cd	13.12 ± 2.17 a	275 ± 0 i	31.2 ± 0.1 ef	4.81 ± 0.12 c	12.08 ± 2.67 e	0.31 ± 0.07 ab	0.78 ± 0.05 c
Jintao	24.04 ± 1.42 c	4.23 ± 0.14 cd	395.83 ± 4.17 e	35.44 ± 0.57 c	4.68 ± 0.97 c	17.46 ± 0.96 d	0.21 ± 0.02 b	0.63 ± 0.05 d
Puyu	17.33 ± 2.56 de	6.07 ± 0.52 c	516.67 ± 8.33 a	60.4 ± 1.36 a	5.01 ± 0.5 c	24.17 ± 0.67 bc	0.26 ± 0.04 a	0.92 ± 0.11 b

The data are given as the mean ± SD (*n* = 3). Mean values in each column with unlike letters are significantly different among cultivars (*p* < 0.05).
